# Enhanced Production of Fatty Acid Ethyl Ester with Engineered *fabHDG* Operon in *Escherichia coli*

**DOI:** 10.3390/microorganisms7110552

**Published:** 2019-11-11

**Authors:** Ziaur Rahman, Bong Hyun Sung, Javed Nawab, Muhammad Faisal Siddiqui, Abid Ali, Almando Geraldi, Sun Chang Kim

**Affiliations:** 1 Department of Microbiology, Abdul Wali Khan University Mardan, Mardan 23200, Khyber Pakhtunkhwa, Pakistan; 2 Synthetic Biology and Bioengineering Research Center, Korea Research Institute of Bioscience and Biotechnology, Daejeon 34141, Korea; bhsung@kribb.re.kr; 3 Department of Environmental Sciences, Abdul Wali Khan University Mardan, Mardan 23200, Khyber Pakhtunkhwa, Pakistan; javednawab11@yahoo.com; 4 Department of Microbiology, Hazara University, Hazara 21120, Khyber Pakhtunkhwa, Pakistan; send2biotech@gmail.com; 5 Department of Zoology, Abdul Wali Khan University Mardan, Mardan 23200, Khyber Pakhtunkhwa, Pakistan; uop_ali@yahoo.com; 6 Department of Biology, Airlangga University, Universitas Airlangga Kampus C, Mulyorejo Surabaya 60115, Indonesia; Almando.geraldi@fst.unair.ac.id; 7 Department of Biological Sciences, Korea Advanced Institute of Science and Technology, Daejeon 34141, Korea; sunkim@kaist.ac.kr

**Keywords:** microbial biofuel, biodiesel, fatty acid synthesis, *Escherichia coli* FAEE, microbial hydrocarbon

## Abstract

Biodiesel, or fatty acid ethyl ester (FAEE), is an environmentally safe, next-generation biofuel. Conventionally, FAEE is produced by the conversion of oil/fats, obtained from plants, animals, and microorganisms, by transesterification. Recently, metabolic engineering of bacteria for ready-to-use biodiesel was developed. In *Escherichia coli*, it is produced by fatty acyl-carrier proteins and ethanol, with the help of thioesterase (TesB) and wax synthase (WS) enzymes. One of the foremost barriers in microbial FAEE production is the feedback inhibition of the fatty acid (FA) operon (*fabHDG)*. Here, we studied the effect of biodiesel biosynthesis in *E. coli* with an engineered *fabHDG* operon. With a basic FAEE producing BD1 strain harboring *tes* and *ws* genes, biodiesel of 32 mg/L were produced. Optimal FAEE biosynthesis was achieved in the BD2 strain that carries an overexpressed operon (*fabH, fabD,* and *fabG* genes) and achieved up to 1291 mg/L of biodiesel, a 40-fold rise compared to the BD1 strain. The composition of FAEE obtained from the BD2 strain was 65% (C10:C2, decanoic acid ethyl ester) and 35% (C12:C2, dodecanoic acid ethyl ester). Our findings indicate that overexpression of the native FA operon, along with FAEE biosynthesis enzymes, improved biodiesel biosynthesis in *E. coli*.

## 1. Introduction

Global warming and scarcity of fossil fuel reserves motivate the scientific community to produce alternative biofuels [1,2,3,4,5]. Biodiesel, or fatty acid ethyl ester (FAEE), is a potential candidate to substitute diesel fuel [6]. Traditionally, it is obtained by transesterification of ethanol with oil/fats obtained from plants, animals, or microorganisms [7]. From an economic point of view, there has been a rise in prices of biodiesel, owing to the use of expensive oil/fats and processing steps [8]. This problem can be overcome by engineering microorganisms that could grow on lignocellulose-derived sugars to produce ready-to-use biodiesel [9]. Wild type *Escherichia coli* is unable to produce FAEE. However, recently, *E. coli* with external thioesterase (TesB) and wax synthase (WS) enzymes produced FAEE from glucose as a carbon source [10]. Some attempts were made that demonstrated the FAEE production in *E. coli* by adding exogenous fatty acids into the culture medium [11,12]. However, the use of exogenous fatty acid in the culture medium is non-economical. Therefore, the in vivo production of FAEE could lower the price of biodiesel [13]. Steen et al. established the FAEE producing pathway in *E. coli* that achieved the titer of 500 mg/L [13]. The custom-designed FAEE pathway in *E. coli* is based on the heterologous expression of thioesterase B (*tesB*) and wax synthase (*ws*) genes. The TesB cleaves fatty acids from acyl carrier proteins (ACP), and the enzyme WS catalyzes trans-esterification of fatty acid to get FAEE [6]. 

### FAEE Production in Bacteria

**Figure 1:**
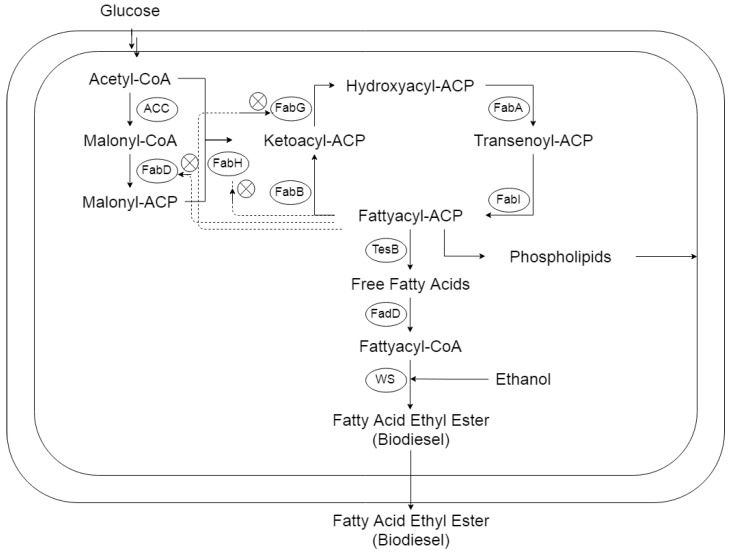
The fatty acid ethyl ester (FAEE) biosynthesis in bacteria. The metabolic pathway of fatty acid synthesis and biodiesel is shown. Enzymes ACC: Acetyl-CoA-carboxylase. FabD: Malonyl-CoA-Synthase. FabH: Malonyl-CoA-ACP-synthase, FabG: Hydroxyacyl-ACP-synthase. FabA: Enoyl-ACP-synthase. FabI: Acyl-ACP-synthase. TesB: Thioesterase. FadD: fatty-acyl-CoA-ligase. WS: wax synthase are shown. The dotted lines represent feedback inhibition.

**Figure 2:**
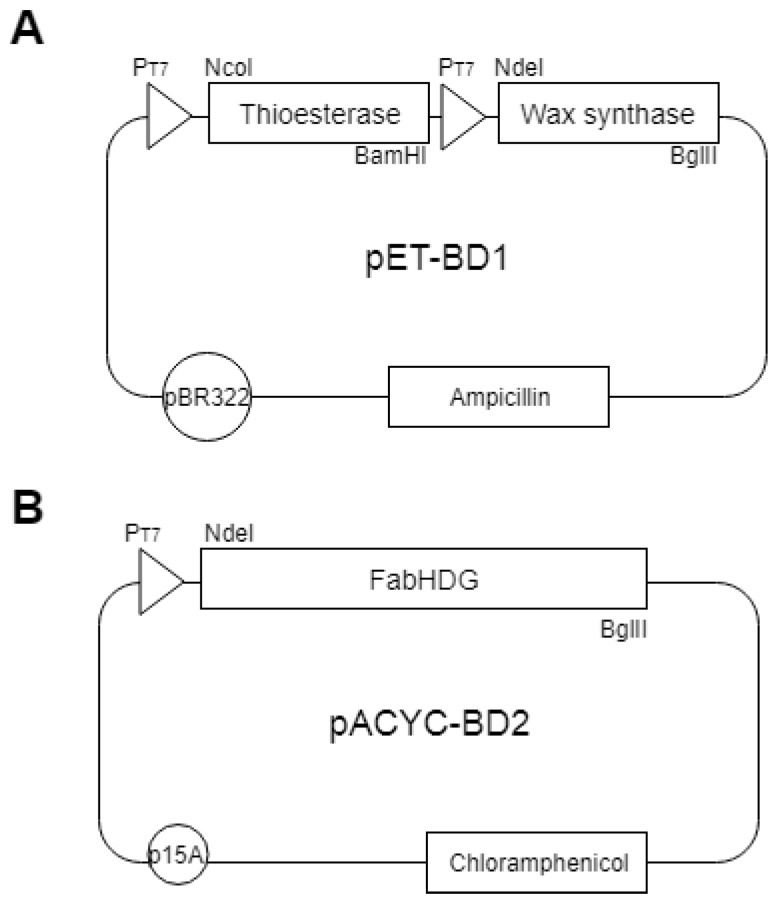
The plasmids. (**A**) pET-BD1 and (**B**) pACYC-BD2.

Fatty acid (FA) production is performed by individual enzymes in a bacterial system [14]. The initiation step of FA is carried out by the acetyl-CoA-carboxylase enzyme, using acetyl-CoA substrate to produce malonyl-CoA. The enzymes of the FA operon (FabD, FabH, FabG) carried out the subsequent FA synthesis steps [14]. The malonyl-CoA is converted to malonyl-ACP by the FabD enzyme (Figure 1). The malonyl-ACP synthase enzyme (FabH) combines acetyl-CoA with malonyl-CoA to make ketoacyl-ACP, an important step in FA synthesis. The elongation step of FA synthesis is performed by multi-enzymes (FabG, FabA, FabI, and FabB), by converting ketoacyl-ACP to hydroxyacyl-ACP, enoyl-ACP, and fatty acyl-ACP (Figure 2) [14,15,16]. The fatty acyl-ACP works as a substrate for phospholipid biosynthesis (Figure 2). 

For FAEE biosynthesis, the TesB enzyme converts fatty acyl-ACPs (FAAs) to free fatty acids (FFAs) [17,18,19,20]. The FFAs are activated and esterified to FAEE by fatty acid-CoA ligase and wax synthase encoded by *fadD* and *ws* genes, respectively [4].

The current production yield of FAEE in bacteria does not meet a commercial threshold. To increase FAEE biosynthesis in *E. coli*, the copy number of native enzymes, encoded by *Acc* and *fabD,* were increased [21]. One of the major problems in biodiesel production is the regulation of the fatty acid pathways in *E. coli* by a strong feedback inhibition mechanism [15,22]. This inhibition is due to elevated levels of fatty acids and the presence of the transcription factor, FadR, to withhold the transcription of the *fab* operon [22,23]. Here, the overexpression of the FA operon (*fabHDG*) in FAEE-producing *E. coli* was studied. The engineered strain with the overexpressed FA operon showed an elevated level of FAEE (biodiesel), up to 40-fold, compared to the control strain. To our knowledge, this is the primary report displaying high productivity of FAEE in *E. coli* by overexpressing the native FA operon in a biodiesel-producing strain. 

## 2. Materials and Methods

### 2.1. Microbial Strains, Enzymes, and Chemicals

The microbial strains, plasmids, and primers are shown in Table 1. *E. coli* strains that were used for expression and production studies were obtained from [24]. Primers were manufactured by GenoTech (Daejeon, Korea). The enzymes and chemicals were purchased from NEB (Beverley, MA, USA) and Sigma-Aldrich (St. Louis, MO, USA), respectively. 

### 2.2. Construction of Plasmids Expressing Tes, and WS

The *tes* gene of *Umbellularia californica* was manufactured by GenoTech corp. (Daejeon, S. Korea). The *ws* gene was PCR-amplified from gDNA of an *Acinetobacter baylyi ADP1* strain, using the respective primers (Table 1). The PCR-amplified DNA fragments of *tes* and *ws* were digested with their respective enzymes and cloned in pETDuet-1 with an ampicillin selection marker (Novagen, Darmstadt, Germany) to obtain pET-BD1 (Table 1, Figure 1). The *E. coli* that was transformed with pET-BD1 was represented as the BD1 strain. 

### 2.3. Construction of Plasmids Expressing the FA Operon (fabHDG)

The FA operon was PCR-amplified from gDNA of *E. coli* strain MG1655 with the respective primers (Table 1). The amplified DNA fragment of the FA operon was digested and cloned in pACYCDuet-1 with a chloramphenicol selection marker (Novagen) to get pACYC-BD2 (Figure 2). The *E. coli* strain was co-transformed with pET-BD1 and pACYC-BD2 to get the BD2 strain. 

### 2.4. Media, Growth Conditions, and FAEE Analysis

LB medium (Tryptone 10 g/L, yeast extract 5 g/L and NaCl 5 g/L) was used for cultivation experiments, according to the method described previously [13,24]. For FAEE production experiments, 2% ethanol was added to the culture media. The antibiotics ampicillin and chloramphenicol were added, 50 and 25 μg/mL, respectively. Bacterial strains were cultured in 500 mL flasks and induction was carried out using IPTG (0.1 mM, OD_600_ = 0.4). The flasks were incubated at 37 °C for 24 h post induction. For FAEE analysis, an equal volume (0.5 mL each) of bacterial culture and chloroform was mixed and vortexed for 60 s, with 5 s intervals. Extraction was carried out with *n*-hexane (0.2 mL) and analyzed by gas chromatography [13,24]. 

## 3. Results and Discussion

### 3.1. FAEE Production in E. coli with Plasmid Encoding TesB and WS

**Figure 3:**
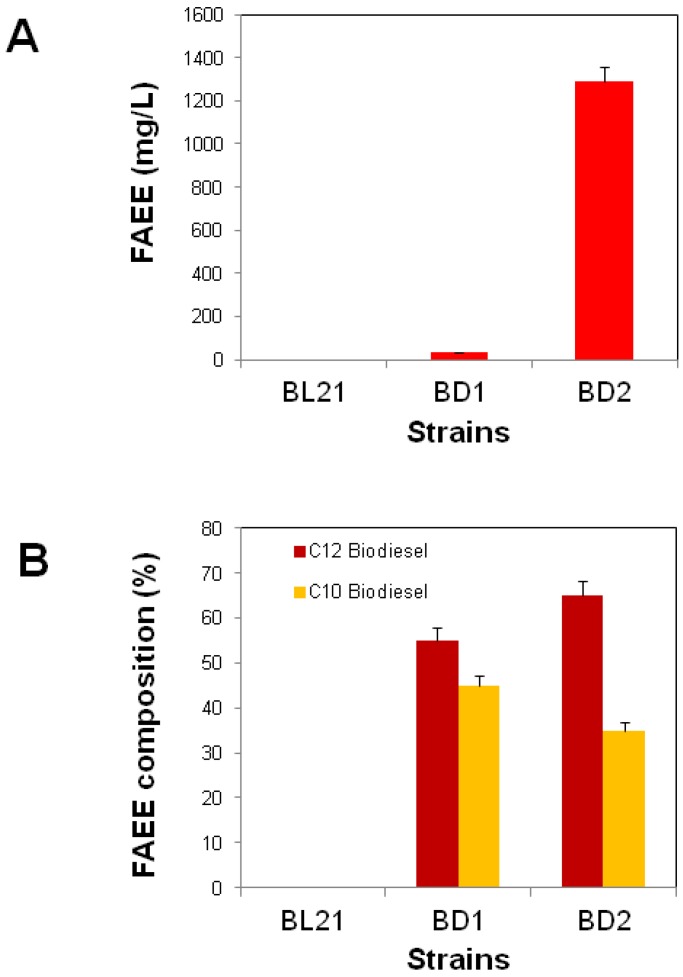
Biodiesel production in engineered *Escherichia coli*. (**A**) Total fatty acid ethyl ester production. (**B**) Composition of fatty acid ethyl ester. Values and error bars represent the mean and standard deviation of triplicate experiments. C12 and C10 biodiesel represent dodecanoic acid ethyl ester and decanoic acid ethyl ester, respectively.

Wild type *E. coli* could not produce biodiesel [11]. The platform strain, with the expression of *tesB* and *ws* genes, has the capability to produce FAEE. The *tesB* and *ws* genes encode for thioesterase B and Wax synthase enzymes that hydrolyze FAAs to FFAs and acyl esterify FFAs, with ethanol, to FAEE, respectively. The BD1 strain harboring pET-BD1 was cultured in 100 mL LB media, with an added 2% glucose and ethanol, and grown at 37 °C (for detail, see Materials and Methods). The BD1 strain produced up to 32 mg/L FAEE after 24 h (Figure 3). This was in agreement with the results of Steen et al., that achieved 37 mg/L FAEE in the strain with minimal FAEE biosynthesis pathway (TesA and Ws) [13]. One of the reasons for the low production of FAEE is because the native fatty acid synthesis enzymes are limited [13], and wild type *E. coli* produces a restricted amount of fatty acids for membrane biosynthesis due to strong feedback inhibition. However, engineered strains with overexpressed thioesterases can produce free fatty acids (a substrate of FAEE biosynthesis) with a theoretical maximum of 0.3–0.4 g/g glucose [26]. This theoretical maximum is not yet achieved for FAEE synthesis due to inhibition of the FA operon [14,27]. The study by Durfe et al. suggested that a stringent response and FAAs minimize the expression of the FA operon [27]. The initiation of FA synthesis is carried out by the FabH, FabD, and FabG enzymes, encoded by the native FA operon, and the inhibition of the operon highly affected FA production (Figure 2). Consequently, the inhibition of the FA operon is also linked with low FAEE yield. Therefore, the effect of overexpression of the FA operon in *E*. *coli*, for FAEE production, was studied and discussed below. 

### 3.2. Improved FAEE Production with the Expression of the FA (fabHDG) Operon

The BD1 strain was transformed with a pACYC-BD2 plasmid, harboring the *fabHDG* operon (FA operon), to get the BD2 strain (see Materials and Methods). The culture conditions for bacterial strains are discussed in Materials and Methods. The BD2 strain was grown with same culture conditions to that of BD1. The BD2 strain, after 24 h, produced FAEE of up to 1291 mg/L. This was 40-fold higher than the BD1 strain (Figure 3). The results suggested that the overexpression of the FA operon improved fatty acid biosynthesis. The enhanced FAEE production (40-fold) with the overexpression of the FA operon suggested the release of feedback inhibition, as supported by previous data [22]. 

The chain length of biodiesel molecules highly affects the quality of biofuel. Biodiesel ranging from C16 to C22 or higher chain lengths adversely affect the combustion and storage properties during cold temperatures [28]. Biodiesel with short chain lengths, of C10 to C14, is considered a good fuel with a greater combustion efficiency and good storage quality. The BD1 and BD2 strains produced short-chain length biodiesel, composed of C10:C2 (decanoic-acid-ethyl-ester) and C14:C2 (tetradecanoic-acid-ethyl-ester). The percentage composition of the BD2 strain was C10:C2 (65%) and C14:C2 (35%), compared to the BD1 strain with C10:C2 (55%) and C14:C2 (45%), as shown in Figure 3. The composition of FAEE could be controlled with different thioesterase enzymes [4]. In previous studies, long chain thioesterase (TesA) was used. With this, a mixture of FAEEs ranging from C14 to C18 was obtained [17]. The overexpression of the FA operon shows promising results, compared to *fabH* overexpression alone [29]. 

A high level of FAEE was obtained in the BD2 strain, via the overexpression of enzymes, without pathway optimization. The titer of FAEE may be enhanced further by increasing the flux towards FA synthesis [30,31], or by lowering the expression of FA regulatory components [32]. Proper balancing of phospholipid and FA biosynthesis pathways may further boost FAEE production by minimizing the stresses in engineered bacterial strains. Furthermore, the FFAs are intermediate in the FAEE pathway and are toxic to *E. coli* [33]. Scaffolding of pathway enzymes for enhanced production of alkanes, by reducing the toxicity of aldehydes in one of our studies, was successful in *E. coli* [24]. Scaffolding of key pathway enzymes, like Tes, FadD, and WS, may enhance FAEE production in *E. coli* towards the commercial threshold.

## Figures and Tables

**Table 1:** Strains, plasmids, and primers.

Items	Description	Reference
Strains		
BL21	*E. coli* str. B F–ompT gal dcm lon hsdSB(rB–mB–) λ (DE3 [lacI lacUV5−T7p07 ind1 sam7 nin5]) [malB+]_K-12_ (λ^S^)	[25]
BD1	BL21 (DE3) + pET-BD1	This study
BD2	BD1 + pACYC-BD2	This study
Plasmids		
pET Duet-1	pBR322 origin.; Amp^r^;	Novagen, Inc.
pACYC Duet-1	p15A origin.; Cm^r^	Novagen, Inc.
pET-BD1	pET Duet-1.; pBR322 origin.; Amp^r^; P_T7_−. (*tesB*) P_T7_ (*ws*)	This study
pACYC-BD2	pACYC Duet-1.; p15A origin.; Cm^r^.; P_T7_− (*fabHDG*)	This study
Primers		
TES-F (*NcoI*)	TTCCATGGATGGCCACCACCTCTTTAGCT	This study
TES-R (*BamHI*)	ATGAGGATCCTTACACCCTCGGTTCTGCGG	This study
WS-F (*NdeI*)	CCAACATATGATGAAGATGAAGAGTTTGATTTA	This study
WS-R (*BglII*)	GGTAAGATCTTTAATTGGCTGTTTTAAT	This study
FAB-F (*NdeI*)	CCAACATATGATGTATACGAAGATTATTGG	This study
FAB-R (*BglI1*)	GGTAAGATCTTTTCAGACCATGTACATCCC	This study
